# Response to Nivolumab and Ipilimumab in Microsatellite Instability**-**High (MSI**-**H) Cervical Carcinoma with Acquired Resistance to Pembrolizumab: A Case Report and Literature Review

**DOI:** 10.1093/oncolo/oyac095

**Published:** 2022-05-28

**Authors:** Gahyun Gim, Yeseul Kim, Yeonggyeong Park, Min Jeong Kim, Myungwoo Nam, Woojung Yang, Samantha E Duarte, Chan Mi Jung, Elena Vagia, Pedro Viveiros, Young Kwang Chae

**Affiliations:** Department of Hematology and Oncology, Wilmot Cancer Institute, Rochester, NY, USA; Department of Internal Medicine, Northwestern University Feinberg School of Medicine, Chicago, IL, USA; Department of Internal Medicine, Northwestern University Feinberg School of Medicine, Chicago, IL, USA; Department of Internal Medicine, Northwestern University Feinberg School of Medicine, Chicago, IL, USA; Department of Internal Medicine, Lincoln Medical Center, New York, NY, USA; Department of Internal Medicine, Northwestern University Feinberg School of Medicine, Chicago, IL, USA; Department of Internal Medicine, Northwestern University Feinberg School of Medicine, Chicago, IL, USA; Department of Internal Medicine, Northwestern University Feinberg School of Medicine, Chicago, IL, USA; Department of Hematology and Oncology, Northwestern University Feinberg School of Medicine, Chicago, IL, USA; Department of Hematology and Oncology, Northwestern University Feinberg School of Medicine, Chicago, IL, USA; Department of Hematology and Oncology, Northwestern University Feinberg School of Medicine, Chicago, IL, USA; Department of Hematology and Oncology, Robert H. Lurie Comprehensive Cancer Center of Northwestern University, Chicago, IL, USA

**Keywords:** anti-PD-1 antibody, anti-CTLA-4 antibody, DNA mismatch repair, MSI-H, immunotherapy

## Abstract

As the use of immune checkpoint inhibitors (ICIs) in treating a variety of cancer types has increased in recent years, so too have the number of reports on patients acquiring resistance to these therapies. Overcoming acquired resistance to immunotherapy remains an important need in the field of immuno-oncology. Herein, we present a case that suggests sequential administration of combination immunotherapy may be beneficial to advanced cervical cancer patients exhibiting acquired resistance to mono-immunotherapy. The patient’s tumor is microsatellite instability-high (MSI-H), which is an important biomarker in predicting ICI response. Results from recent interim prospective studies using combination immunotherapy (eg, nivolumab and ipilimumab) with anti-PD-1 plus anti-CTLA-4 inhibitor following progression on anti-PD-1 inhibitors (eg, nivolumab) have shown anti-tumor activity in patients with advanced melanoma and metastatic urothelial carcinoma. We also introduce retrospective studies and case reports/case series of dual checkpoint inhibition with anti-PD-1 inhibitor plus anti-CTLA-4 inhibitor after progression on prior anti-PD/PD-L1 monotherapy. To date, there has been no prospective study on the use of combined anti-PD-1 and anti-CTLA-4 therapy at the time of progression on anti-PD-1 therapy in patients with MSI-H tumors or advanced cervical cancer. In this report, we provide evidence that supports future investigations into such treatments.

## Teaser

As immune checkpoint inhibitor therapy has become more common, an increasing number of patients have shown acquired resistance to such therapies. This case report suggests that sequential administration of combination immunotherapy may be beneficial for patients with advanced cervical cancer exhibiting acquired resistance to mono-immunotherapy.

**Key Points**

- This case report is believed to be the first demonstrating clinical efficacy of dual immunotherapy after acquired resistance to pembrolizumab in a patient with MSI-H status cervical carcinoma.
- The patient showed a notable response to dual combination therapy with nivolumab and ipilimumab for more than 2 years.

## Introduction

The recent expansion of research in the field of cancer genomics has allowed immunotherapy to become the new treatment paradigm. Microsatellite instability (MSI) is now being used as an important biomarker to guide immunotherapy.^1^ Microsatellites are defined as short, repeating DNA sequences that are prone to slippage during DNA replication. Under normal conditions, these replication errors can be repaired by the DNA mismatch repair (MMR) system. Defects in MMR (dMMR) proteins can lead to an accumulation of mutations in microsatellite sequences resulting in a state known as MSI. Microsatellite mutations located within coding sequences can produce abnormal proteins involved in DNA repair, apoptosis, or cell growth, which can ultimately lead to the development of cancer.^2^

Although neoantigens from MSI-H tumors can elicit an immune response, MSI-H tumors also express checkpoint receptors (eg, PD-1, CTLA-4) at higher rates, which suppress the immune response and immune-mediated tumor lysis.^3^ Therefore, blocking PD-1 using anti-PD-1 antibodies can restore anti-tumor immune activity. In May 2017, the U.S. Food and Drug Administration (FDA) granted approval to pembrolizumab for use in patients with MSI-H/dMMR solid tumors that showed progression after prior treatment.^4^ Notably, this is the first cancer treatment approved by the FDA to be based solely on biomarker status, rather than on the anatomical site of cancer.

However, even with a significant initial response to immunotherapy, patients are at risk for developing acquired resistance to immunotherapy and subsequent progression of the disease.^5^ Therefore, overcoming acquired resistance to immunotherapy remains a high unmet need in the field of immuno-oncology. Herein, we present the first case of advanced cervical cancer with MSI-H status that showed a notable response to dual combination immunotherapy with nivolumab and ipilimumab after acquired resistance to pembrolizumab.

## Patient Story

A 60-year-old woman with a history of former smoking (5-pack per day) and negative human papillomavirus (HPV) status presented with a chief complaint of bloody vaginal discharge lasting 1 month. Pelvic examination was notable for a large friable lesion in the central cervix. A cervical biopsy performed demonstrated poorly differentiated invasive squamous cell carcinoma. A positron emission tomography-computed tomography (PET-CT) showed a large fluorodeoxyglucose (FDG)-avid cervical mass invading the uterine body without any evidence of remote metastasis. She was then diagnosed with stage IB1 squamous cell carcinoma of the cervix. Concurrent chemoradiation therapy was initiated with 45 Gy in 25 fractions and then weekly cisplatin infusions, followed by high dose rate brachytherapy.

**Figure 1.**
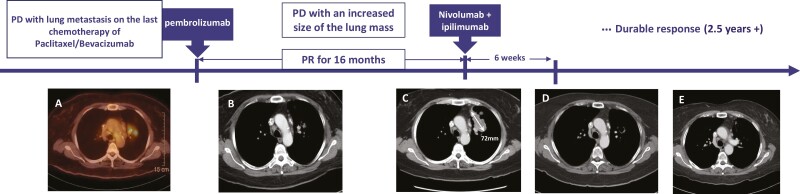
Serial images of a patient with MSI-H cervical carcinoma receiving nivolumab and ipilimumab. (A) PET-CT after 2 months of paclitaxel and bevacizumab. (B) CT scan throughout 16 months of pembrolizumab. (C) CT scan at 16 months of pembrolizumab treatment, showing disease progression with an increased size of a lesion measuring 72 mm. (D) CT scan after 6 weeks of nivolumab and ipilimumab, confirming a deep partial response with a 10 mm-sized cystic lesion. (E) CT scan after 2.5 years of dual immunotherapy.

After 6 months, the patient received extended-field radiation therapy with concurrent cisplatin chemotherapy for 1 month to treat the newly detected peri-aortic lymph node chains. Surveillance PET-CT acquired 2 months after the treatment revealed resolution of the para-aortic lymphadenopathy. However, imaging also demonstrated FDG uptake by a new 1.1 cm-sized left perihilar nodule. PET-CT acquired 5 months later showed enlargement of the left perihilar mass (up to 4.0 cm) and numerous hypermetabolic pulmonary nodules. Therefore, the decision was made to change the systemic therapy regimen to paclitaxel and bevacizumab. PET-CT acquired after 2 months of treatment with paclitaxel and bevacizumab showed a marked interval decrease in the degree of metabolic activity and size of the left perihilar mass, as well as an interval decrease in the size and number of the pulmonary nodules, consistent with partial response (RECIST 1.1) (Fig. 1A). She continued treatment with paclitaxel and bevacizumab combination therapy.

However, the follow-up PET-CT at 4 months revealed a new focus of FDG uptake in the anterior left upper lobe with stable left perihilar mass. Increased metabolic activity was observed in retroperitoneal lymph nodes, and there was local recurrence in the left aspect of the cervix. Therefore, the treatment regimen was changed to topotecan. However, this therapy was discontinued after a month due to symptomatic anemia and thrombocytopenia requiring transfusion.

## Molecular Tumor Board

Circulating tumor DNA assay by next generation sequencing (Guardant Health, Redwood City, CA) revealed *PIK3CA* E542Q mutation (1.8%, variant allele frequency). FoundationOne (Foundation Medicine, Inc.; Cambridge, MA) results showed TMB-high (24 Muts/Mb) and MSI-high/dMMR. PD-L1 expression on either tumor or immune cells (Tumor proportion score, Combined positive score) by immunohistochemistry (DAKO antibody 22C3 clone) showed negative expression (Tempus; Chicago, IL).

Based on her MSI-H status, the patient was started on immunotherapy with pembrolizumab 200 mg once every 3 weeks. She had a partial response followed by the stable disease throughout the 16 months of pembrolizumab treatment (Fig. 1B). At 16 months however, she was found to have disease progression with an increase in the size of the left upper lobe mass (Fig. 1C). Given her tolerability of immunotherapy, she started on a dual immunotherapy with nivolumab 3 mg/kg once every 2 weeks and ipilimumab 1 mg/kg once every 6 weeks to possibly overcome immunotherapy resistance. After 6 weeks, the follow-up CT scan demonstrated a deep partial response (RECIST 1.1). The left lung mass decreased in size significantly from 72 mm to a 10-mm-sized cystic lesion (Fig. 1D). Other pulmonary subcentimeter nodules remained stable.

## Patient Update

Follow-up CT scans revealed a fibrotic change of the lesion and showed an ongoing durable response for more than 2 years (Fig. 1E). She has experienced immune-related toxicities including acute pancreatitis, colitis, and adrenal insufficiency, all of which have been resolved completely. To date, she has good adherence to the dual immunotherapy and reported no other symptoms.

## Discussion

To our knowledge, this is the first reported case of a patient with advanced cervical cancer with MSI-H status that showed a notable response to dual combination immunotherapy with nivolumab and ipilimumab after acquired resistance to an anti-PD-1 inhibitor. Cervical cancer is the fourth most common cancer in women globally, with most cases associated with HPV infection.^6^ Recently, pembrolizumab has been added as a second-line systemic therapy regimen for recurrent cervical cancer after the KEYNOTE-158 study demonstrated the safety and antitumor activity of pembrolizumab in multiple solid tumors (NCT02628067). However, current therapeutic options are limited for recurrent cervical cancer that develops acquired resistance to anti-PD-1 inhibitors. Overcoming resistance to immunotherapy is, therefore, a high unmet need currently. Our case highlights the potential benefit of administering sequential combination immunotherapy to patients with advanced cervical cancer or any solid tumor with MSI-H status exhibiting acquired resistance to anti-PD-1 inhibitors.

There are currently 10 ongoing prospective studies exploring the safety and efficacy of combination immunotherapy with patients who progressed on single-agent anti-PD-1 inhibitors **[**Table 1**]**. Four of these studies have released interim results demonstrating overall tolerability and anti-tumor activity in patients with NSCLC, advanced melanoma, and metastatic urothelial carcinoma. In one study, patients with NSCLC who either had primary or acquired resistance to prior anti-PD-1 or anti-PD-L1 monotherapy, then received durvalumab IV 20 mg/kg every 4 weeks and tremelimumab IV 1 mg/kg every 4 weeks. The interim results showed an objective response rate (ORR) of 2/38 (5.3%) in the primary resistant group and 2/40 (5%) in the acquired resistant group. The study also reported a disease control rate (DCR) of 8 (21.1%) in the primary resistant group and 9/40 (22.5%) in the acquired resistant group. Both ORR and DCR are similar among patients with primary and acquired resistance (NCT02000947). Another study of 17 melanoma patients revealed a high response rate with 4 cycles of pembrolizumab and ipilimumab followed by pembrolizumab monotherapy after progression on any of the FDA-approved single-agent anti-PD-1 monotherapy. This study reported an ORR of 8/17 (47%) and DCR of 13/17 (76%) (NCT02743819). In a study on metastatic urothelial carcinoma, 21 patients received a combination therapy of 4 cycles of nivolumab and ipilimumab following progression on nivolumab monotherapy. The interim result showed an ORR of 4/21 (19%) (NCT02553642). Also, studies have shown the positive results of tolerability of using combined immunotherapy. In the NSCLC study group, a total of 28% of patients had grades 3-4 immune-related adverse events (AEs) and 14% of grades 3-4 AEs were reported in the melanoma group (NCT02000947, NCT02743819). In the metastatic urothelial carcinoma group, relatively high grades 3-4 AEs were reported at 43% compared with the other 2 studies. However, despite the high number of patients with grades 3-4 AEs in metastatic urothelial carcinoma, 44% of those with grades 3-4 AEs patients had disease control for more than 3 months, which suggests that the benefit of dual immunotherapy possibly outweigh the potential risk of developing high-grade AEs (NCT02553642). Lung Cancer Master Protocol or Lung MAP evaluates both the targeted therapy and immunotherapy in squamous NSCLC. In the sub-study arm F (S1400F) of Lung MAP trial, 30 patients received 4 cycles of durvalumab and tremelimumab combination therapy. Both durvalumab and tremelimumab target PD-L1 and CTLA-4, respectively. Patients were previously treated with ≥24 weeks of anti-PD-(L)1 monotherapy and developed disease progression. The interim results showed an ORR of 0/30 (0%) and DCR of 14/30 (47%). The dual immunotherapy targeting both PD-L1 and CTLA-4 had no association with objective response. However, it showed clinically meaningful DCR of 47%. A total of 33% of patients experienced grades 3-4 AEs.

**Table 1.** Clinical trials evaluating efficacy of dual-immunotherapy with anti-PD-1/PD-L1 inhibitors plus anti-CTLA-4 inhibitors after an acquired resistance to anti-PD-1/PD-L1 inhibitors.

Cancer type	Eligibility criteria	Primary outcome	Dosing regimen	Results	Reported irAEs	Phase/enrollment number	Status	Clinical trials identification number
Advanced NSCLC	Primary resistant (group A) or acquired resistant (group B) to prior anti-PD-1 or anti-PD-L1 monotherapy	AEs, objective response (CR, PR, or PD according to RECIST 1.1), DLTs	DUR IV 20 mg/kg Q4W for up to 12 months + TREM IV 1 mg/kg Q4W with the first 4 cycles of DUR	Interim result 38 of primary resistant (group A), 40 of acquired resistant (group B) patients received treatment (total 78) -Confirmed ORR: 2 (5.3%) in A, 2 (5%) in B (total 4; 5.1%) -DCR at 24 weeks: 8 (21.1%) in A, 9 (22.5%) in B (total 17; 21.8%) -Median PFS: 1.7 months in A, 2.5 months in B -Median OS: 8.3 months in A, 8.5 months in B	AEs reports in 72% of patients -Grade 3/4 AEs in 28% of patients -5 patients (6%) discontinued due to AEs -no treatment-related death occurred	Phase I/ 459	Completed	NCT02000947
Advanced melanoma	Patients who had PD or SD lasting at least 24 weeks during treatment with an anti-PD-1 monotherapy	Response rate as assessed by irRECIST	PEM 200 mg + IPI 1 mg/kg q3w for 4 doses, followed by PEM alone	Interim result -17 response-evaluable patients accrued -CR 2/17 (11.8%), PR 6/17 (35.3%) – ORR 47% -SD 5/17 (29.4%) – DCR 76% -PFS at 6 months: 75%	Any AEs reports in 14/22 (64%) -Grade 3-4 AEs in 3 patients (14%)	Phase II/ 70	Recruiting	NCT02743819
Metastatic urothelial carcinoma	Platinum resistant mUC patients who had PD with NIVO monotherapy	Response rate based on RECIST 1.1 criteria	(IPI 3 mg/kg + NIVO 1 mg/kg) IV Q3W for 4 doses	Interim result 21 response-evaluable patients accrued -CR 0, PR 4/21 (19%) – ORR 19% -SD 6/21 (28.6%) – DCR 47.6% -PD 11/21 (51%)	-Grade 3-4 AEs in 9 patients (43%) -4/9 of grade 3-4 AEs (44%) had disease control (PR + SD > 3 months)	Phase II/ 81	Active, not recruiting	NCT02553642
Advanced renal cell carcinoma	Patients who had PD on NIVO maintenance after induction with NIVO and IPI	DCR	(NIVO + IPI) IV q3w for 4 doses	NA	NA	Phase II/ 100	Recruiting	NCT04088500
Recurrent or metastatic head and neck squamous cell carcinoma	Patients who were previously exposed to an anti-PD-1 or PD-L1 monotherapy	Incidence of AEs according to CTCAE v. 4.0	DUR IV + TREM IV q4w for up t 4 courses or q6w for up to 3 courses, then DUR IV q4w for up to 9 courses or q6w for up to 6 courses. Patients also undergo hypofractionated radiation therapy over 3 fractions QOD during week 3	NA	NA	Phase II/ 20	Recruiting	NCT03522584
Locally advanced unresectable or metastatic basal cell carcinoma	Patients who had PD on an anti-PD-1 monotherapy	ORR based on RECIST 1.1 criteria	IPI 1 mg/kg IV q4w for 4 doses + NIVO 480 mg IV q4w followed by NIVO 480 mg IV q4w for up to 48 total weeks of therapy	NA	NA	Phase II/ 40	Recruiting	NCT03521830
Recurrent stage IV lung cancer	Patients who had PD on prior platinum-based chemotherapy; Patients also must had PD on an anti-PD-1 or anti-PD-L1 monotherapy	ORR by RECIST	(DUR + TREM) IV q4w	Interim result 30 response-evaluate patients accrued -ORR 0/30 (0%) -SD 14/30 (47%) -Median PFS: 2.0 months -Median OS: 7.5 months	-Grade 3-4 AEs in 10/30 (33%) -1treatment-related death occurred due to pneumonitis and 1 death not otherwise specified	Phase II/ 132	Recruiting	NCT03373760 Lung-MAP trial
Anti-PD-1-axis therapy-resistant advanced NSCLC	Primary resistant or acquired resistant to prior anti-PD-1 monotherapy	ORR by RECIST 1.1	NIVO 3 mg/kg q2w + IPI 1 mg/kg IV q6w	NA	NA	Phase II/ 50	Recruiting	NCT03262779
Stage IV or stage III unresectable melanoma	Patients with advanced melanoma refractory to an anti-PD-1 or anti-PD-L1 monotherapy	PFS	(NIVO + IPI) IV q3w for up to 4 cycles followed by NIVO IV q4w	NA	NA	Phase II/ 94	Recruiting	NCT03033576
Stages III–IV melanoma progressed or relapsed on PD-1 inhibitor therapy	Patients who had PD on an anti PD-1 or PD-L1 monotherapy	Objective response by RECIST v 1.1 criteria	(NIVO IV 1 mg/kg + IPI IV 3 mg/kg) q3w for 4 doses	NA	NA	Phase II/ 19	Completed	NCT02731729

Abbreviations: NSCLC, non–small cell lung cancer; AEs, adverse events; DLTs, dose-limiting toxicities; CR, complete response; PR, partial response; PD, progressive disease; ORR, overall response rate; DCR, disease control rate; PFS, progression-free survival; OS, overall survival; PEM, pembrolizumab; IPI, ipilimumab; RR, response rate; SD, stable disease; mUC, metastatic urothelial carcinoma; NIVO, nivolumab; QxW, every (x) weeks; DUR, durvalumab; TREM, Tremelimumab; NA, not available; CTCAE, common terminology criteria for adverse events; RT, radiation therapy; QOD, every other day.

Retrospective studies and case reports have described patients who received anti-PD-1 inhibitor plus anti-CTLA-4 inhibitor after progression on prior anti-PD/PD-L1 monotherapy (Table 2). Three retrospective studies on patients with advanced melanoma showed the efficacy of dual immunotherapy with a high DCR rate of 33%, 47.4%, and 60%, respectively. Two of these studies demonstrated OSR of 68.5% and 55% respectively. There are 4 advanced melanoma case reports on a total of 5 patients treated with nivolumab and ipilimumab dual combination immunotherapy after progression on prior anti-PD-1/PD-L1 monotherapy. Three patients had PR after 3 to 4 doses of the dual treatment. One patient had a mixed response at 8 weeks, followed by a durable response of up to 6 months. Another patient had a metabolic response with a decline in uptake from PET-CT scan and down trending tumor marker for 6 months.

**Table 2.** Retrospective studies and case reports or case series of dual checkpoint inhibition with anti-PD-1 inhibitor plus anti-CTLA-4 inhibitor after progression on prior anti-PD/PD-L1 monotherapy.

Cancer type	Detailed information	Regimen/dose	Result	Reported irAEs
Metastatic melanoma(Gaughan, Petroni, Grosh,and Slingluff, 2017)	Metastatic melanoma patients with NIVO/IPI combination therapy after progression on prior anti-PD-1 or PD-L1 monotherapy. Patients had received up to 4 lines of prior immunotherapy	NIVO + IPI	Total 19 patients. ORR 2/19 (10.5%), DCR 9/19 (47.4%). 6 months survival 68.5%.	8 patients stopped treatment due to toxicities. 13 patients had clinically significant toxicities (rash, colitis, hepatitis, and hypophysitis)
Metastatic melanoma (Mehmi & Hill, 2018)	Patients who were treated with IPI and anti-PD1 therapy (NIVO or PEMBRO) after PD with first-line anti-PD-1 monotherapy	IPI + Anti-PD-1 inhibitor (NIVO or PEMBRO)	Total 15 patients. CR 5/15 (33%), PR or SD 4/15 (27%), DCR 60%.	5 patients discontinued IPI/anti-PD-1 due to grade 3-4 adverse events (dermatitis, encephalitis, and nephritis).
Advanced melanoma (Zimmer et al, 2017)	Advanced melanoma patients who were treated with NIVO + IPI after treatment failure to anti-PD-1 monotherapy	NIVO (1 or 3 mg/kg) + IPI (1 or 3 mg/kg)	Total 37 patients. ORR 21%, DCR 33%, one-year OSR 55%	NA
Metastatic melanoma (2 patients) (Ugurel et al, 2017)	Patients with NIVO/IPI combination therapy after progression on prior NIVO 3 mg/kg 4 doses	NIVO 1 mg/kg + IPI 3 mg/kg q3w for 4 times	Initial worsening of symptoms and parameters (LDH, S100B, and CRP). But these parameters decreased rapidly after 2 and 3 doses of combination therapy and symptoms improved subsequently. Imaging demonstrated PR after 4 doses in both patients.	NA
Metastatic melanoma (Glutsch et al, 2019)	Prior pembrolizumab was discontinued after a single dose for developing AKI with nephrotic syndrome due to MCD. The patient was found to have PD after resolution of AKI and started combination therapy	NIVO 1 mg/kg + IPI 3 mg/kg	A deep partial remission (RECIST 1.1) after 3 doses of NIVO + IPI. LDH peaked after the first dose of combination therapy and then trended down. S-100 level decreased with the course of combination therapy.	NA
BRAF-mutant advanced melanoma with brain metastasis (Spain, Schmid, Gore, and Larkin, 2017)	BRAF-mutant melanoma. Patient was treated with NIVO + IPI following primary progression on pembrolizumab after 4 cycles	NIVO + IPI (+ dexamethasone for symptomatic brain edema)	Mixed response after 8 weeks. After 6 months, the patient had a maintained response. But PD at 7 months after the initiation of combined therapy.	Immune-related hepatitis, managed with escalation of dexamethasone and initiation of mycophenolate mofetil.
Metastatic colon cancer and localized urothelial cancer (Winer et al, 2019)	Lynch syndrome patient with metastatic colon cancer, later found to have localized urothelial cancer. Absent/lacking MSH-2 and MSH-6 expression in both tumors. The patient was treated with PEMBRO for 9 mo, ATEZO for 8 mo until PD prior to dual-immunotherapy	NIVO 1 mg/kg + IPI 3 mg/kg q3w for 4 cycles followed by NIVO 3 mg/kg q4w	CEA peaked right after the first dose of combination therapy and down trended. Imaging revealed decline in uptake in metastatic colon cancer and shrinkage of urothelial tumor	After 7 months of continued disease control with the combination immunotherapy, bilirubin rose to 5.3, which was treated with high-dose steroids

Abbreviation: NIVO, nivolumab; IPI, ipilimumab; ORR, overall response rate; DCR, disease control rate; OSR, overall survival rate; NA, not available; CR, complete response; PR, partial response; SD, stable disease; PD, progression of disease; irAEs, immune-related adverse events; QxW, every (x) weeks; LDH, lactate dehydrogenase; S100B, S100 calcium-binding protein B; CRP, C-reactive protein; NA, not available; AKI, acute kidney injury; MCD, minimal change disease; PEMBRO, pembrolizumab; ATEZO, atezolizumab.

In this report, we present the first case demonstrating the effectiveness of combination immunotherapy in MSI-H tumors that failed a single-agent anti-PD-1 inhibitor. The importance of our finding stems from the patient’s response to different immunotherapy following progression on 2 other anti-PD-1 inhibitors. This patient developed metastatic colorectal cancer and urothelial cancer. He was diagnosed with Lynch syndrome, an inherited form of MMR deficiency given the lack of MSH-2 and MSH-6 protein expression in both tumors. The patient was subsequently treated with pembrolizumab, atezolizumab, and finally combination immunotherapy with nivolumab 3 mg/kg and ipilimumab 1 mg/kg every 3 weeks for 4 cycles followed by nivolumab monotherapy 3 mg/kg every 4 weeks. The patient experienced a durable response to the dual combination immunotherapy over 7 months.

The efficacy of combination immunotherapy in the setting of prior resistance to single-agent anti-PD-1/PD-L1 inhibitors varies among different cancer types. For example, the prospective study on melanoma demonstrated a notably higher DCR of 76% relative to the DCR of NSCLC and metastatic urothelial carcinoma (21.8% and 19%, respectively). In addition, the DCR varied from 33% to 60% in the retrospective studies on a melanoma group, remaining higher than the DCR of NSCLC and metastatic urothelial carcinoma as reported in the prospective studies. These reports suggest that melanoma has the highest response rate among the 3 histologies. Therefore, in the clinical setting, combination immunotherapy should be considered in treating melanoma patients who failed a single-agent anti-PD-1 inhibitor.

The results of this study have motivated us to identify a group of patients who might also benefit from combination immunotherapy to overcome acquired resistance to anti-PD-1 therapies. The prospective and retrospective studies have shown the antitumor effects of using combination immunotherapy after acquired resistance to mono-immunotherapy, although these studies have focused on tumors with high tumor mutational burden (TMB). This proof-of-concept has been shown mainly in the 3 tumor types (melanoma, lung, and bladder) which are known to be immunogenic tumors harboring high TMB. Therefore, we speculate that the concept of combination therapy in patients resistant to mono-immunotherapy can be generalized to other tumors that have high TMB and/or MSI-H. We expect that patients with MSI-H tumors may experience meaningful clinical benefits from the combination immunotherapy.

To date, we are not aware of any ongoing clinical trials exploring the use of dual anti-PD-1 and anti-CTLA-4 therapy at the time of progression on anti-PD-1 inhibitors in patients with MSI-H tumors. Our group is designing a prospective clinical trial of dual-immunotherapy in patients with MSI-H tumors who have progressed on anti-PD-1 inhibitors to support the efficacy of using combined immunotherapy. Such further investigations are warranted to better understand the role of dual immunotherapy in the setting of immune resistance.

## Data Availability

The data underlying this article will be shared on reasonable request to the corresponding author.
